# Concreteness training reduces dysphoria: A pilot proof-of-principle study

**DOI:** 10.1016/j.brat.2008.10.014

**Published:** 2009-01

**Authors:** Edward R. Watkins, Nicholas J. Moberly

**Affiliations:** Mood Disorders Centre, School of Psychology, University of Exeter, Exeter EX4 4QG, United Kingdom

**Keywords:** Cognitive training, Rumination, Overgeneralization, Depression, Concrete

## Abstract

We hypothesized that a tendency towards abstract, general and decontextualized processing is a cognitive distortion that causally contributes to symptoms of depression. This hypothesis predicts that training dysphoric individuals to become more concrete and specific in their thinking would reduce depressive symptoms. To test this prediction, participants with stable dysphoria (scoring ≥ 14 on BDI-II at 2 consecutive weekly assessments) were randomly allocated in an additive design either to an active intervention control consisting of relaxation training or relaxation training plus concreteness training. Concreteness training involved repeated mental exercises designed to encourage more concrete and specific thinking about emotional events. Both interventions involved a training session and then repeated daily use of compact disc recordings for 7 days. Relaxation training plus concreteness training resulted in significantly greater decreases in depressive symptoms and marginally significantly greater decreases in state rumination than relaxation training alone. These findings suggest the potential value of concreteness training as a guided self-help intervention for mild-to-moderate depressive symptoms.

## Introduction

Major and minor depression are highly prevalent disorders, affecting approximately 10–20% of the population. Moreover, there is a recognised difficulty in accessing psychological treatments (Scogin, Hanson, & Welsh, 2003), reflecting a shortfall in the number of trained therapists relative to the prevalence of depression. Therefore, recent expert panels and governmental policy have recommended that the development of effective and easily disseminable interventions for depression is a priority (e.g., Hollon et al., 2002; Layard, 2004). One effective and easily disseminable intervention is guided self-help (Gellatly et al., 2007). To date, self-help packages have focused on psychoeducation and teaching patients cognitive-behavioural techniques. However, despite cognitive models emphasizing the role of cognitive biases in depression (Beck, 1976; Clark, Beck, & Alford, 1999), no self-help intervention has tried to directly modify these cognitive biases. Here we report the first pilot test of direct cognitive bias modification as guided self-help for individuals with depressive symptoms.

One cognitive bias strongly implicated in the onset and maintenance of depression is the tendency to process self-relevant information in an overgeneralized and abstract manner (Beck, 1976). For example, a single negative event, such as a failure, is interpreted as indicating a global, characterological inadequacy (Carver & Ganellen, 1983). Overgeneralization prospectively predicts subsequent levels of depression (Carver, 1998; Dykman, 1996; Edelman, Ahrens, & Haaga, 1994). Depression is also characterized by increased recall of overgeneral memories, characterized by categoric summaries of repeated events (e.g., “making mistakes”), when asked to recall specific personal memories (Williams et al., 2007). Again, overgeneral memory retrieval predicts poorer long-term outcome for depression in prospective studies (Williams et al., 2007).

This tendency towards overgeneralization parallels the conceptualization of abstract construals within the social-cognitive literature (e.g., Trope & Liberman, 2003; Vallacher & Wegner, 1987). Abstract construals are general, superordinate and decontextualized mental representations that convey the essential gist and meaning of events and actions, such as inferences of global traits that are invariant across different situations (e.g., “laziness”), or representations of “why” an action is performed and of its ends and consequences. In contrast, concrete construals are mental representations that include subordinate, contextual, and incidental details of events and actions, such as inferences of situation-specific states, such as “tiredness”, or representations of the specific “how” details of an action and of the means to an end. Thus, depression involves a cognitive bias characterized by the abstract construal of (particularly negative) self-relevant actions and events.

Moreover, this *abstract-overgeneral cognitive bias* mediates the effect of failure on subsequent negative affect (Brown & Dutton, 1995; Kernis, Brockner, & Frankel, 1989; Wenzlaff & Grozier, 1988). Further, practising recall of specific autobiographical memories reduces the negative response to a later stressful task relative to recall of general autobiographical memories (Raes, Hermans, Williams, & Eelen, 2006).

A recent approach to testing the causal role of cognitive biases in psychopathology is to train participants towards or away from a cognitive bias by repeated practice on cognitive-experimental tasks (e.g., Mathews & Mackintosh, 2000; Wilson, MacLeod, Mathews, & Rutherford, 2006). This *cognitive bias modification* (CBM) paradigm has demonstrated that training participants to adopt concrete construals (“imagine the details of what is happening”) when thinking about emotional scenarios reduces emotional reactivity to a subsequent failure manipulation, relative to training in adopting abstract construals (“think about the causes, meanings and implications”; Moberly & Watkins, 2006; Watkins, Moberly, & Moulds, 2008). Thus, level-of-construal causally influences emotional reactivity, with more abstract construals resulting in greater emotional reactivity.

However, these studies only involved a mild failure induction and non-clinical participants low in symptoms of depression, raising the question of the generalizability of these findings to real-world settings and more dysphoric samples. Furthermore, the training only consisted of a single 30 min experimental session and the durability of this brief training on emotional reactivity is not known. Nonetheless, if as hypothesized, level-of-construal does causally influence emotional reactivity, sustained changes in level-of-construal would be expected to influence depressive symptoms. Thus, a logical conclusion of these findings is that training individuals to adopt more concrete construals (henceforth *concreteness training*, CNT) might have potential as a guided self-help intervention for depression. The current study was designed to test “*proof-of-principle*” with respect to whether repeated CNT is an intervention capable of reducing naturally occurring depressive symptoms in a dysphoric sample. To increase the likelihood of a stable and durable effect of concreteness training, the training involved repeated daily training over a week.

We used a matched active intervention control – progressive muscle relaxation training (RT) – as a comparison to the CNT condition in an *additive* design (i.e., RT vs. RT + CNT). We chose this design for three reasons. First, because relaxation is an active intervention shown to have a moderate effect size for reducing depressive symptoms (Murphy, Carney, Knesevich, Wetzel, & Whitworth, 1995; Reynolds & Coats, 1986), it provides a more conservative test of the efficacy of CNT, relative to the waiting-list control typically used in the first evaluation of novel treatments. Second, this additive design reduces the likelihood that any differential treatment response was due to positive expectations, demand effects or other non-specific therapy factors. Third, because relaxation has no treatment component focused on changing cognition, the additive design tested whether the specific cognitive components of CNT add any treatment benefit to the relaxation components (i.e., whether CNT works by processes other than relaxation). We predicted that the addition of CNT to RT would produce significantly greater decreases in depressive symptoms from pre-training to post-training than RT alone.

Further, depressive rumination is characterized by recurrent thinking about the *causes, meanings and implications* of depressive symptoms (Nolen-Hoeksema, 1991), and is thus characterized by abstract construal. Moreover, experimental studies have indicated that abstract construals play a causal role in the pathological effects of rumination on autobiographical memory, self-judgements and problem-solving (Rimes & Watkins, 2005; Watkins & Moulds, 2005; Watkins & Teasdale, 2001). Watkins (2008) summarized evidence indicating that abstract construal is an important dimension in the development of depressive rumination and hypothesized that shifting people away from abstract construals would reduce depressive rumination. Thus, we predicted that RT + CNT would produce significantly greater decreases in state rumination than RT alone.

## Method

### Participants

We tested participants with stable dysphoria, operationalized as individuals with scores of at least 14 on the BDI-II (i.e., above the cut-off for mild depression; Beck, Steer, & Brown, 1996) at two consecutive weekly assessments (baseline and pre-training assessments). This multiple-gating procedure ensures that level of depressive symptoms is relatively stable and that participants are actually dysphoric at the time of intervention. It also reduces error variance and the likelihood that any observed improvements are due to spontaneous recovery (Ingram & Siegle, 2002; Kendall, Hollon, Beck, Hammen, & Ingram, 1987). In total, 39 participants (21 female and 7 male students, 9 female and 2 male community adults) met this criterion and completed the training intervention week and final assessment. We arrived at our final sample-for-analysis after initially recruiting 75 dysphoric adults (22 males) with scores of at least 14 on the BDI-II using email and newspaper advertisements. Of these 75 participants, 10 failed to return for the pre-training assessment, 20 no longer scored at least 14 on the BDI-II at the pre-training assessment, and 5 participants withdrew from the study at the pre-training assessment expressing concerns over time commitments. One further participant (in the CNT condition) dropped out of the study during the training week because she was unable to practise the exercises. The six participants who dropped out of the study did not differ significantly from participants who completed the study on any of the baseline measures or demographic variables. Following the pre-training assessment, the 39 participants meeting criteria were randomized to the RT + CNT vs. RT interventions.

### Materials

#### RT

Participants were initially given a rationale for the relaxation exercises which explained that the exercises were designed to be helpful for relieving worry and stress. Participants were then led through a 12 min guided progressive muscle relaxation procedure in which they practised tensing and relaxing various parts of their body while focusing on their breathing and bodily sensations. Finally, participants were given a 12 min compact disc (CD) recording of the relaxation exercises and were asked to practise relaxing while listening to the CD at a convenient time each day. At this point, the beneficial properties of the relaxation exercises were reiterated. The total relaxation training procedure lasted approximately 20 min.

#### RT + CNT

Participants were initially given a rationale for the concrete thinking training exercises which explained that the exercises were designed to be helpful for relieving worry and stress. Participants were then led through the guided relaxation procedure as described above. Next, participants were guided through mental exercises on several scenarios to practise inducing more concrete construals. For example, participants were asked to read a printed description of a hypothetical incident in which the participant arrived at work late and was then asked a difficult question by their boss. These scenarios were phrased to encourage the participant to adopt a first-person perspective when imagining the event (i.e., a field perspective; Nigro & Neisser, 1983). For the practice scenarios and for all subsequent training scenarios, participants were given the following concreteness instructions: “Focus on how the event happened and imagine in your mind as vividly and concretely as possible a ‘movie’ of how the event unfolded. As you imagine the event, see it through your own eyes, from your own viewpoint, as if you are looking out on the scene. Imagine the event in the present tense, as if you are there right now.” After 1 min spent imagining the scenario, participants were asked a series of questions asking them to focus on and describe what they could see, hear, feel, what actions they were performing and how the event unfolded moment to moment (e.g., “What can you see in this event? How does the event unfold moment-by-moment?”). During this process, the experimenter questioned the participant to ensure that they provided detailed, sensory-oriented responses.

Participants practised this mental exercise for 30 more hypothetical scenarios printed on cards (15 positive, e.g., winning a competition; 15 negative, e.g., being in a car accident), using the same concreteness instructions. Participants read a prompt card listing the questions focusing attention on sensory details of their experience. Participants were given 1 min to read and imagine each scenario, before they were handed the next card. After 10 and 20 scenarios, the experimenter read out the list of prompt questions before continuing. As a further element of CNT, participants were then asked to remember and imagine for 1 min (i) a positively valenced autobiographical memory, (ii) a negatively valenced autobiographical memory, and (iii) an autobiographical memory of a specific event in which they experienced a sense of flow (Csikszentmihalyi, 1990), all with the same concreteness instructions. In this way, CNT was extended from hypothetical events to personal memories, thereby encouraging participants to use the concrete style when contemplating self-experience. For the flow memory, participants were told to recollect an “experience in which you were totally absorbed and immersed in the process and details of what you were doing, when you were so mentally involved in an activity that you lost all sense of time and all self-consciousness.” Because flow experiences correspond to occasions when individuals are exclusively focused on their current activity and environment, they reflect experiences in which concrete thinking dominates. Such recall should therefore enhance CNT.

CNT (including the relaxation exercises) lasted approximately 70 min. At the end of this period, participants were given a CD recording of the exercises to practise at home. The CD included two tracks, each lasting approximately 30 min. Each track replicated the sequence and contents of the training session (in order): relaxation exercise; imagining 15 imaginary scenarios for 1 min each using the concreteness instructions; imagining a positively valenced autobiographical memory, a negatively valenced autobiographical memory and an autobiographical memory for a flow experience, for 1 min each using the same concrete, sensory-focused thinking style. The recording prompted participants to think of different memories each time they practised the exercise. The experimenter asked the participant to practise these exercises by listening to one of the CD tracks at a convenient time each day for seven days, changing the track they listened to each day.

#### Beck Depression Inventory-II (BDI-II; Beck et al., 1996)

The BDI-II assesses levels of depressive symptomatology with 21 items that are each rated on a scale from 0 to 3, with higher scores reflecting more depressive symptoms (range 0–63). We adapted the BDI-II so that participants indicated how they were feeling over the *last week*. Cronbach's alpha for our sample was .83 at the initial baseline assessment, .81 at the pre-training assessment, and .85 at the post-training assessment.

#### Response Styles Questionnaire–Ruminative Responses Scale (RSQ; Nolen-Hoeksema & Morrow, 1991)

The RSQ measures the extent to which individuals respond to depressed mood by focusing on self, symptoms and on the causes, meanings and consequences of their mood, with 22 items that are each rated on a 4-point frequency scale. The RSQ items also capture level of abstract construal, given their focus on meanings, understanding and thinking about “Why?” (e.g., Think “Why do I always react this way?”). Cronbach's alpha for our sample at the initial baseline assessment was .87. We included a modified ‘state’ version of the RSQ in which the wording of each item was adapted so that participants were asked to report on the frequency with which they had ruminated *over the last week*. Cronbach's alpha for this adapted RSQ was .73 at the pre-training assessment and .86 at the post-training assessment.

### Procedure

At an initial briefing session, participants provided informed consent and completed the RSQ and BDI-II (Assessment 1: baseline). An appointment was then made with the participant to attend again at least one week later. Upon returning for this phase, participants completed the BDI-II and state RSQ (Assessment 2: pre-training). If participants continued to meet the criteria for dysphoria, they were then randomized into either the RT condition or the RT + CNT condition. Seven days after the training session, the experimenter contacted the participant and asked whether he or she had been able to practise the exercises seven times. If not, the participant was asked to continue practising until this condition had been met. Participants were contacted twice weekly until they reported that they had practised the exercise seven times, at which point an appointment was made for the final assessment. On this occasion, participants again completed the BDI-II and state RSQ (Assessment 3: post-training) before being paid and debriefed.

## Results

### Background characteristics

For the analysed sample (see below), we examined whether demographic (gender, age, status) and baseline variables (BDI-II, RSQ) differed between conditions by conducting a multivariate ANOVA with Training Condition (RT + CNT vs. RT) as the between groups factor (see Table 1). This revealed no significant difference between conditions, *F*(5, 30) < 1.

### Training procedure

Participants in the RT + CNT condition took significantly more days (*M* = 15.6, SD = 8.7) to practise the exercises seven times and return for the final assessment than participants in the RT condition (*M* = 11.1, SD = 4.3), *t*(37) = 2.08, *p* < .05. This was due to three outliers (2 male students, 1 female student) in the RT + CNT condition who took, respectively, 30, 33, and 38 days to practise the training exercises seven times and return for the final assessment. When these participants were removed from the dataset, there was no longer any significant difference in training interval between RT + CNT (*M* = 12.2, SD = 3.3) and RT (*M* = 11.1, SD = 4.3), *t*(34) = 1.14, *p* = .39. These participants are not included in analyses reported below, but their inclusion did not change the pattern of significant findings.

### Effect of training on depressive symptoms

**Fig. 1:**
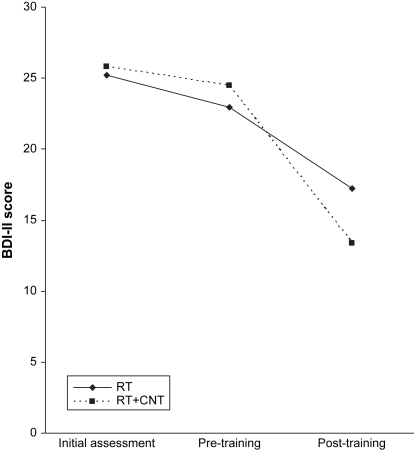
Depressive symptomatology at each assessment by condition.

Table 2 provides descriptive statistics for the BDI-II before and after training (Assessments 2 and 3). To test our prediction that RT + CNT would reduce depressive symptoms significantly more than RT, we conducted a mixed-design 2 × 2 ANOVA, with Training Condition (RT + CNT vs. RT) as the between groups factor, Time (pre-training vs. post-training) as the repeated measures factor, and with BDI-II score as the dependent variable. As predicted, there was a significant main effect of time, *F*(1, 34) = 79.72, *p* < .001, partial *η*^2^ = .70, reflecting a reduction in depressive symptoms across the training phase for both conditions, which was qualified by a significant Condition × Time interaction, *F*(1, 34) = 8.13, *p* < .01, partial *η*^2^ = .19. This interaction is illustrated in Fig. 1, which clarifies that the Condition × Time interaction reflected a greater decrease in depressive symptoms pre- to post-intervention in RT + CNT, *t*(15) = 6.76, *p* < .001 (95% CI: 7.62, 14.63), than in RT, *t*(19) = 5.41, *p* < .001 (95% CI: 3.52, 9.67).

### Effect of training on state RSQ scores

Table 2 provides descriptive statistics for the state RSQ before and after training (Assessments 2 and 3). We predicted that RT + CNT would reduce tendency towards abstract construals, and thereby reduce levels of depressive rumination, to a significantly greater extent than RT alone. To test this prediction we conducted a mixed-design 2 × 2 ANOVA, with Training Condition (RT + CNT vs. RT) as the between groups factor, Time (pre-training vs. post-training) as the repeated measures factor, and with state RSQ as the dependent variable. There was a significant main effect of time, *F*(1, 34) = 24.22, *p* < .001, partial *η*^2^ = .42, reflecting a reduction in state rumination across the training phase for both conditions. Importantly, this main effect was qualified by a marginally significant Condition × Time interaction, *F*(1, 34) = 3.83, *p* < .06, partial *η*^2^ = .10, such that state rumination reduced more from pre- to post-intervention in RT + CNT than in RT (see Table 2).^1^

## Discussion

The current study aimed to test “*proof-of-principle*” with respect to the prediction that CNT has value as an intervention for symptoms of depression. We found that although both RT and RT + CNT conditions were associated with significant decreases in depressive symptoms, these reductions were significantly greater for participants in the RT + CNT condition than for participants in the RT condition. Thus, CNT had added treatment benefit in reducing depressive symptoms above and beyond relaxation, itself an active treatment for depression. Furthermore, the trend towards a greater decrease in depressive rumination for RT + CNT relative to RT provides some evidence that CNT specifically reduced thinking characterised by abstract construals (Watkins, 2008).

We found that repeated practice at thinking about emotional situations in a more concrete and specific way reduced depression and rumination to a greater extent than relaxation alone. These results provide initial “proof-of-principle” concerning the potential causal role of level-of-construal in depression, extending (a) previous longitudinal studies finding that overgeneral and abstract processing prospectively predicted depression and (b) previous experimental studies finding that abstract, overgeneral processing influenced emotional vulnerability to stressful tasks.

This study was intended as a preliminary investigation of the value of translating CNT from a single-session experimental manipulation into a repeated clinical intervention. The comparison with relaxation, as an active treatment control, reduces the possibility that the benefits from CNT were solely due to expectancy and demand effects. The additive design means that the effects of CNT are not fully reliant on the relaxation component of the intervention. Thus, these results indicate that concreteness training passes the first pilot tests necessary to establish that an experimental paradigm may be translatable into a useful clinical intervention: (a) the training was able to influence ongoing naturally occurring depressive symptoms; (b) the training was able to have an impact in a more vulnerable, at-risk population (participants with mild-to-moderate depression scores on the BDI-II).

The levels of depression pre-training were characteristic of those seen by general practitioners working in primary care, suggesting that this intervention may have value as an adjunct to existing interventions for depression in primary care. Further, if the treatment effects can be replicated, CNT could be easily delivered as a guided self-help intervention, with the patient undertaking the training exercises with minimal guidance from a health professional. This format would mean that the training would potentially be relatively inexpensive to administer, and highly accessible and disseminable. Nonetheless, before concluding that CNT is an efficacious clinical intervention, we need (a) to further examine the durability and stability of its antidepressant effect and (b) to examine its generalizability to patients with diagnoses of depression. We are currently conducting clinical trials to ascertain the generalizability of CNT to patients with diagnoses of major depression.

There are several limitations to this study. First, the duration of the treatment effect was not examined beyond one week. Nonetheless, the levels of depressed symptoms were within the mild-to-moderate range and remained stable across two assessments, suggesting that this sample provided a reasonable sub-clinical analogue. Second, we did not assess whether the intervention changed diagnostic status. Third, the sample consisted of a dysphoric sample rather than patients with a diagnosis of major depression. Fourth, all our measures were self-report: a more conservative and rigorous study would include observer and interviewer-rated measures of depression. Fifth, we did not assess the mechanisms of change in detail: there was no measure of change in level-of-construal other than state rumination, leaving open the possibility that some other element within the concreteness training was responsible for its beneficial effect. The state rumination measure was also adapted from the RSQ and has not been psychometrically validated. Future research could examine more specific mediators of symptom change. Sixth, the duration of RT + CNT was longer than that of RT, a necessary consequence of testing whether CNT provides added therapeutic value beyond relaxation. However, self-focused thinking is not necessarily beneficial (Nolen-Hoeksema, 1991) such that increased time thinking about the self (in CNT) would not be expected to be beneficial unless it contained some specifically therapeutic elements. Further, as a predominantly self-delivered intervention, the discrepancy in duration is less important than in therapist-delivered interventions, where non-specific factors such as support, empathy and understanding could all contribute to treatment effects. Nonetheless, the increased duration of CNT and/or the increased contact time with the experimenter during the initial training session could account for its more significant treatment effects. Future studies could usefully equate the conditions for practice and contact time. Alternatively, CNT could be compared with an equal-duration bogus training condition, matched for rationale and duration but lacking its active ingredients, to unpack the contribution of shared non-specific factors. Furthermore, after receiving the initial training and rationale, participants could rate their expectancies about the treatment benefits associated with each condition. A recently completed study compared concreteness training vs. an equal-duration bogus training condition matched for rationale, vs. a waiting-list control condition in a dysphoric sample (Watkins, Baeyens, & Read, in press). This study found that concreteness training was a more efficacious intervention than the other conditions, increasing our confidence that CNT may have a genuine therapeutic effect.

Despite these limitations, we have demonstrated that it is possible to reduce level of depressive symptoms in dysphoric individuals by repeated practice in adopting a more concrete way of processing self-related information, relative to a relaxation intervention. To our knowledge, these results are the first demonstration that attempts to directly modify cognitive biases via self-help interventions can influence depressive symptoms. These findings are consistent with the hypothesis that abstract, overgeneral thinking is a cognitive bias that causally contributes to depressive symptoms, although this hypothesis was not directly tested. These findings also suggest that there may be value in further developing and evaluating concreteness training as a potential self-help intervention for mild-to-moderate depression.

## Figures and Tables

**Table 1:** Sample characteristics and means (SDs) at the first assessment by condition.

Variable	Condition	
	RT (*n* = 20)	RT + CNT (*n* = 16)
Gender	15 females	13 females
Status	14 students	11 students
Age	28.7 (14.0)	29.6 (16.0)
Baseline BDI-II	25.2 (7.0)	25.8 (9.7)
Baseline RSQ	62.4 (10.5)	59.4 (11.2)
Baseline RSQ brooding	14.6 (3.7)	14.2 (3.1)
Baseline RSQ reflection	13.1 (3.6)	12.3 (2.8)

*Note*: BDI-II = Beck Depression Inventory-II; RSQ = Response Style Questionnaire–Rumination subscale; RT = relaxation training; RT + CNT = relaxation training plus concreteness training.

**Table 2:** Means (SDs) for depressive symptomatology and state rumination before and after training.

Measure	Condition	
	RT (*n* = 20)	RT + CNT (*n* = 16)
BDI-II		
Pre-training	23.0 (6.1)	24.5 (8.1)
Post-training	17.2 (8.0)	13.4 (8.9)

State RSQ		
Pre-training	49.2 (8.4)	49.5 (5.9)
Post-training	44.9 (10.2)	39.5 (8.2)

*Note*: BDI-II = Beck Depression Inventory-II; State RSQ = Response Style Questionnaire–Rumination subscale modified to assess the past week; RT = relaxation training; RT + CNT = relaxation training plus concreteness training.

## Footnotes

1 When we analysed the brooding subscale of the RSQ separately (Treynor, Gonzalez, & Nolen-Hoeksema, 2003), the pattern of results was extremely similar to that of the full RSQ. Thus, there was a significant main effect of Time, *F*(1, 34) = 12.04, *p* = .001, partial *η*^2^ = .26, and a significant Time by Condition interaction, *F*(1, 34) = 5.87, *p* = .02, partial *η*^2^ = .15. However, for the reflection subscale of the RSQ, there was a significant drop from pre-training to post-training, *F*(1, 34) = 6.11, *p* = .02, partial *η*^2^ = .15, but no significant Time by Condition interaction.
